# Limited Vitrectomy in Patients with Idiopathic Macular Hole

**DOI:** 10.4274/balkanmedj.galenos.2019.2018.12.103

**Published:** 2019-10-28

**Authors:** Berna Özkan, Veysel Levent Karabaş, Büşra Yılmaz Tuğan, Özgül Altıntaş

**Affiliations:** 1 Department of Ophthalmology, Acıbadem Mehmet Ali Aydınlar University School of Medicine, İstanbul, Turkey; 2 Department of Ophthalmology, Kocaeli University School of Medicine, Kocaeli, Turkey; 3 Clinic of Ophthalmology, İzmit Seka State Hospital, Kocaeli, Turkey

**Keywords:** Idiopathic, macular hole, retinal breaks, retinal detachment, vitrectomy

## Abstract

**Background:**

Partial posterior hyaloidectomy is suggested to minimize traction on the vitreous base and thus reduce the risk of iatrogenic breaks in patients with macular hole and epiretinal membrane.

**Aims:**

To evaluate the safety and efficacy of limited vitrectomy in patients with macular hole.

**Study Design:**

Retrospective cohort study.

**Methods:**

Fifty-two consecutive patients who underwent macular hole surgery without complete peripheral vitreous removal were included in the study. The improvement in visual acuity, the incidence of retinal breaks and detachment, anatomical results, and intraoperative and postoperative complications of this technique were evaluated.

**Results:**

The median visual acuity was 0.2 (0.1-0.4) before surgery and 0.5 (0.3-0.6) after surgery (p<0.001). None of the patients had retinal breaks or detachments. A sulfur hexafluoride was used in 24 patients (46.2%), and perfluoropropane was used in 28 patients (53.8%). Three patients (5.76%) had revision surgery because of recurrence of the macular hole. We did not observe proliferative vitreoretinopathy or surgery-related major complications in any patient during the follow-up period.

**Conclusion:**

Limited vitrectomy without removal of the peripheral vitreous seems to be effective and safe with minimal risk of peripheral retinal breaks and detachment.

Idiopathic macular holes are full-thickness retinal defects in the neurosensory foveal retina. The classical approach to macular hole surgery consists of pars plana vitrectomy, internal limiting membrane (ILM) peeling, gas tamponade, and face-down positioning. Classic pars plana vitrectomy requires complete removal of the vitreous, including the vitreous base. However, the aim of macular hole surgery is to remove tangential and anteroposterior vitreous traction, induce marginal glial cell activity by vitrectomy and epiretinal membrane peeling and reconstruct hole edges by intraocular gas tamponade (1). All of these procedures are performed in the macular area.

In macular surgeries such as macular hole and epiretinal membrane, we routinely do not perform a complete vitrectomy. We believe that this method might reduce complications such as iatrogenic retinal breaks, and it does not affect the anatomical and functional results. The aim of our study is to evaluate the safety of limited vitrectomy in patients with macular hole.

## MATERIALS AND METHODS

The records of patients who underwent vitrectomy for idiopathic macular hole were reviewed retrospectively. The follow-up visits were at the 1^st^ day, 1^st^ week, 1^st^ month, 3^rd^ month, 6^th^ month, and 12^th^ month. This study evaluated the 12^th^ month examination results. The Ethics Committee of Kocaeli University approved the study (Date: 27.12.2017 No: KÜ GOKAEK 2017/362). Informed constent was taken from all study participants.

### Surgical technique

Vitrectomy was performed with standard 23-gage instruments (OS4, Oertli Instrumente AG, Berneck, Switzerland), and an Oculus BIOM non-contact viewing system (Oculus Surgical, Port St. Lucie, FL, USA) was used on all patients. After core vitrectomy, posterior vitreous detachment (PVD) was performed, and the posterior hyaloid was removed. The PVD was advanced to the equator but not further. The vitreous in front of the equator was trimmed, but it was not completely removed. Then, ILM was stained with 0.025% Brilliant Blue G 250 over the macular region. After a 20-second exposure, the dye was aspirated with a back-flush needle. The ILM was peeled around the macular hole across the macula for the whole area within the arcade using end-gripping ILM forceps. If we observed peripheral retinal degeneration during the surgery, we applied prophylactic laser photocoagulation around the degeneration. Then, fluid air exchange was performed. One of the trocars was removed, and the closure of the sclerotomy was checked. A sulfur hexafluoride (SF6) or perfluoropropane (C3F8) gas line was attached to the infusion line, and a back-flush was placed to the other trocar. After the gas was injected into the vitreous cavity, the rest of the trocars were removed.

If cataracts were observed in the preoperative evaluation, a combined phacoemulsification and intraocular lens implantation was also planned. Combined cataract surgery was performed with phacoemulsification before the vitrectomy procedure. Following insertion of an anterior chamber maintainer, a side-port was created from 10 o’clock for the right eye and 2 o’clock for the left eye. A continuous curvilinear capsulorrhexis was created with a cystotome. Then, a 2.5-mm limbal corneal tunnel was created from the steep region of the patient’s corneal keratometric measurement. Phacoemulsification (EasyPhaco, OS4, Oertli Instrumente AG, Berneck, Switzerland) was followed by aspiration of cortical remnants. The intraocular lens used in all eyes was a 3-piece hydrophobic acrylic intraocular lens (Sensar, Acrylic Intraocular Lens AR40e; Abbott Medical Optics, Inc. Santa Ana, CA, USA). At the end of the cataract surgery, a viscoelastic solution was left in the anterior chamber. Then, we proceeded to pars plana vitrectomy in order to keep the anterior chamber stabilized during the surgery. The viscoelastic solution was removed at the end of vitrectomy before fluid air exchange.

The patients were positioned face down for 3 days. Follow-up visits were scheduled at the 1^st^ day, 1^st^ week, 1^st^ month, 3^rd^ month, 6^th^ month, and 1^st^ year. If the macular hole persisted after surgery, a revision surgery was performed. In revision surgery, we did not remove the residual vitreous. Following placement of 23-gage trocars, 0.025% Brilliant Blue G 250 was poured over the macula to visualize the remaining ILM. Then, liquid perfluorocarbon was injected to cover the whole macular area in order to manipulate the ILM flap and graft and close the hole. Recurrent holes were closed in three ways. In the first patient, the ILM was peeled in a small area in the previous surgery, and an ILM flap was created and inverted over the macular hole to close it. In the second patient, an ILM graft was harvested and tucked inside the well of the macular hole. In the third patient, we advanced the edges of the hole by massage and aspirated the subretinal fluid from the well of the hole with 41 G backflush under perfluorocarbon. This third maneuver usually helps us to close the hole during surgery. In revision surgery, SF6 or C3F8 was used as the tamponade in all cases.

### Follow-up examinations

In preoperative and postoperative follow-up examinations, patients’ best corrected visual acuity, intraocular pressure, biomicroscopy, and fundus were evaluated. Additionally, optical coherence tomography (Heidelberg Engineering GmbH, Heidelberg, Germany) was performed at all visits.

### Statistical analysis

All statistical analyses were performed using IBM SPSS for Windows version 20.0 (SPSS, Chicago, IL, USA). We performed a post hoc analysis, and the power of the study was calculated as 92% based on intraocular pressure with an alpha error of 0.05 and with an effect size of 0.48 using G*Power 3.1.9.2 (Kiel University, Kiel, Germany) software. The assumption of normality was assessed by using a one-sample Kolmogorov-Smirnov test. Normally distributed continuous variables were expressed as mean ± standard deviation, and non-normally distributed continuous variables were expressed as median (25^th^-75^th^ percentile). Normally distributed continuous variables between groups (regarding gas groups) were compared by using the Student’s t-test. For non-normally distributed continuous variables, differences (between results before and after surgery) were tested by using a Wilcoxon signed rank test. A two-sided p value <0.05 was considered to indicate statistical significance.

## RESULTS

Fifty-two patients with macular holes were evaluated. Twenty-seven (51.91%) of the patients were male, and 25 (48.07%) of them were female. The mean patient age was 62.02±12.05 (Table 1).

The median visual acuity was 0.2 (0.1-0.4) before surgery, and it was 0.5 (0.3-0.6) 12 months after surgery. Visual acuity was significantly higher post-operatively than pre-operatively (p<0.001). The mean intraocular pressure was 15.71±3.77 mmHg before surgery and 14.12±2.56 mmHg 12 months after surgery. Intraocular pressure was also significantly lower post-operatively than pre-operatively (p=0.023).

Twenty-two (42.3%) patients had combined phacoemulsification and intraocular lens implantation. There was no difference in final visual acuity between patients who had only vitrectomy and those who had the combined procedure (p=0.610). We did not observe any major complications related to the additional operation. Only two patients had posterior synechiae of the iris, which did not affect visual acuity (3.84%). The eyes were filled with gas at the end of the surgery. SF6 (14% gas) was used in 24 patients (46.2%), and C3F8 (14% gas) was used in 28 patients (53.8%). None of the patients had a retinal break during surgery. We did not observe retinal tears, retinal detachment or proliferative vitreoretinopathy in any patients during follow-up. Three patients (5.76%) had revision surgery for recurrence of the macular hole. These holes were closed successfully after reoperation.

## DISCUSSION

Iatrogenic retinal break is one of the most serious complications of macular hole surgery. Retinal breaks may occur because of existing peripheral retinal degeneration or traction of the retina by vitreous during surgery. This traction may be created by vitreous incarceration into the sclerotomy site or accidental vitreous traction during instrumentation. Rizzo et al. (2) reported a similar incidence of post-vitrectomy retinal detachment in small-gage vitrectomy compared with 20-gage surgery. On the other hand, some studies reported a higher incidence of retinal breaks with 20-gage than with 23-gage vitrectomy (3,4). These studies suggested that the incidence of these two traction causes may be reduced by use of the 23-gage or 25-gage trocar systems (5). Today, most surgeons are using trocar systems in macula surgeries.

Another reason for vitreous traction during surgery is to separate the vitreous from the retina and advance it up to the vitreous base. If there is a focal area of vitreoretinal adhesion, applying stress to this adhesion may increase the risk. Rahman et al. (6) reported that that rate of iatrogenic retinal breaks associated with posterior hyaloid face separation during 23-gage pars plana vitrectomy was 18.2%. They concluded that mechanical detachment of the posterior hyaloid increases the risk of rhegmatogenous retinal detachment and is an important risk factor in the formation of retinal breaks. Chung et al. (7) reported that the rate of retinal breaks related to macular hole surgery was 14.6%, and they detected postoperative retinal detachment in 2.2% of their patients. They commented that pushing forward the PVD to the peripheral retina would force traction on the posterior vitreous base margin, which may cause retinal breaks in any orientation to the retinal meridian. We believe that leaving the peripheral vitreous would decrease the risk of retinal breaks in macular hole surgery. In the current study, we did not perform complete removal of the peripheral vitreous, and none of our patients had retinal breaks during surgery or retinal detachment during follow-up.

Limited vitrectomy has been proposed in a few recent studies. Kim et al. (8) suggested that partial posterior hyaloidectomy could decrease the incidence of traction on the vitreous base and minimize the risk of iatrogenic breaks in patients with macular hole and epiretinal membrane. Their technique is to remove as much of the vitreous gel as possible before PVD. The PVD was induced by engaging the posterior vitreous with a 23G needle with an angulated tip. However, the PVD was limited to a distance of approximately two-disk diameters beyond the margin of the temporal major vascular arcade. In our study we conducted PVD after core vitrectomy, and then we trimmed the anterior vitreous without extending the vitreous base. Although the sequences of PVD were different, both studies aimed to reduce traction in the vitreous base. In agreement with our results, they reported that the rate of retinal break formation was significantly lower compared with conventional 23G surgery. They found retinal breaks related to surgery only in two eyes (3.4%) during postoperative examination. Both of their patients were in the macular hole surgery group. Cullinane and Cleary (9) performed posterior hyaloid peeling only in the macular area. Retinal tears were not observed in this group during the surgery, and two (3.6%) patients had postoperative retinal detachment. They found a similar postoperative retinal detachment rate (4%) in the conventional vitrectomy group. Additionally, they observed intraoperative retinal tears in five (6%) patients in the conventional vitrectomy group.

PVD has been shown to be correlated with the incidence of retinal breaks (7,10). A significantly higher postoperative retinal break rate was reported in patients with macular hole than in those with epiretinal membrane (7,11). Chung et al. (7) explained that vitreous architecture differed between patients with macular hole and those with epiretinal membrane. The reported prevalence of PVD is 57%-90% in patients with idiopathic epiretinal membrane and 20%-40% in those with macular hole (11,12,13). They observed PVD in 78.5% of patients with epiretinal membrane and in 23.4% of patients with MH (7). The authors suggested that surgeons must be cautious because of the accompanying retinal breaks that occur with PVD induction. Tan et al. (14) also reported that retinal breaks were found more often in eyes in which PVD was induced during surgery (20.8%) compared with the incidence in eyes in which PVD was already present at the beginning of the surgery (10.0%). It may be hypothesized that attached posterior vitreous might be continuous with the attached peripheral vitreous, and attempting to advance the detachment might cause unnecessary traction on the peripheral retina. This might lead to an additional risk of retinal breaks.

Kamizuru and Ogura (15) showed that lattice degeneration was seen more often in eyes with macular hole than in controls. In another study, the incidence of lattice degeneration was found to be 33.88% in patients with macular holes (16). Yagi et al. (10) evaluated the relation between iatrogenic retinal breaks and the lattice degeneration frequency in patients with macular hole and epiretinal membrane. They revealed lattice degeneration in 17.7% (14/79 eyes) in the macular hole group and 9.8% (4/41 eyes) in the epiretinal membrane group. The rate of retinal breaks was 71.4% in those with lattice degeneration in the macular hole group. They concluded that the rates of lattice degeneration and retinal breaks were significantly higher in eyes with macular hole (10). Since lattice degeneration is a risk factor for iatrogenic retinal breaks, avoiding traction in the lattice degeneration area might guard against retinal breaks. In our study, we did not remove the vitreous over these peripheral retinal degenerations, and we applied prophylactic laser photocoagulation around that area. We did not observe retinal breaks or postoperative retinal detachment in any of our patients.

We used intraocular gas tamponade in order to maintain immobilization and apposition of the hole edges in all patients. One might think that residual vitreous might induce contraction and cause postoperative retinal breaks or retinal detachment. However, we did not observe these complications, as stated earlier. Another problem with leaving the peripheral vitreous might be inadequate filling of the gas tamponade. However, the amount of gas was sufficient to close the hole in most of our patients. Only three patients had recurrent macular holes after surgery. The macular holes in these patients were closed successfully after revision surgery.

Recently, authors have advocated that using long-lasting tamponades with pars plana vitrectomy may achieve closure of the macular hole without face-down positioning (17,18,19,20). They claim that patients who undergo macular hole surgery are usually old and have comorbidities that reduce compliance with posturing. It has also been reported that the success of this technique was reduced in macular holes larger than 400 μm. We recommended a face-down position to all our patients. The effectiveness of limited vitrectomy with and without face-down positioning may also be compared.

In our study, we did not compare our patients’ results with those of a control group composed of patients with complete vitrectomy. We only presented our technique and compared the results with the literature. This may be a weakness of our study. Comparing our technique with a conventional control group might strengthen our results.

In conclusion, limited vitrectomy without removal of the peripheral vitreous seems to be  effective and safe with minimal risk of peripheral retinal breaks and  detachment.

## Figures and Tables

**Table 1:**
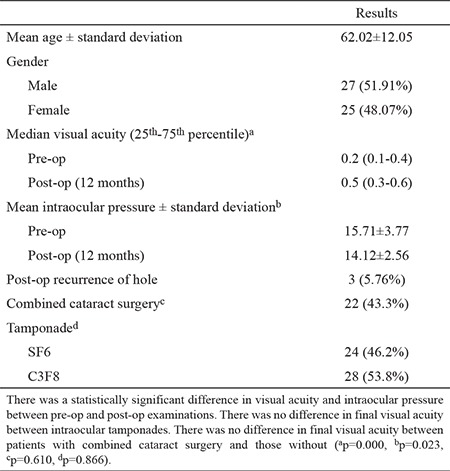
Clinical and surgical characteristics of the patients

## References

[1] Chin EK, Almeida DR, Sohn EH (2014). Structural and Functional Changes After Macular Hole Surgery: a review. Int Ophthalmol Clin.

[2] Rizzo S, Belting C, Genovesi-Ebert F, di Bartolo E (2010). Incidence of retinal detachment after small-incision, sutureless pars plana vitrectomy compared with conventional 20 gauge vitrectomy in macular hole and epiretinal membrane surgery. Retina.

[3] Krishnan R, Tossounis C, Fung Yang Y (2013). 20-gauge and 23-gauge phacovitrectomy for idiopathic macular holes: comparison of complications and long-term outcomes. Eye (Lond).

[4] Issa SA, Connor A, Habib M, Steel DH (2011). Comparison of retinal breaks observed during 23 gauge transconjunctival vitrectomy versus conventional 20 gauge surgery for proliferative diabetic retinopathy. Clin Ophthalmol.

[5] Nakano T, Uemura A, Sakamoto T (2011). Incidence of iatrogenic peripheral retinal breaks in 23-gauge vitrectomy for macular diseases. Retina.

[6] Rahman R, Murray CD, Stephenson J (2013). Risk factors for iatrogenic retinal breaks induced by separation of posterior hyaloid face during 23-gauge pars plana vitrectomy. Eye (Lond).

[7] Chung SE, Kim KH, Kang SW (2009). Retinal breaks associated with the induction of posterior vitreous detachment. Am J Ophthalmol.

[8] Kim JH, Kang SW, Kim YT, Kim SJ, Chung SE (2013). Partial posterior hyaloidectomy for macular disorders. Eye (Lond).

[9] Cullinane AB, Cleary PE (2000). Prevention of visual field defects after macular hole surgery. Br J Ophthalmol.

[10] Yagi F, Takagi S, Tomita G (2014). Incidence and causes of iatrogenic retinal breaks in idiopathic macular hole and epiretinal membrane. Semin Ophthalmol.

[11] Guillaubey A, Malvitte L, Lafontine PO, Hubert I, Bron A, Berrod JP, et al (2007). Incidence of retinal detachment after macular surgery: a retrospective study of 634 cases. Br J Ophthalmol.

[12] Hirokawa H, Jalkl AE, Takahashi M, Takahashi M, Trempe CL, Schepens CL (1986). Role of the vitreous in idiopathic preretinal fibrosis. Am J Ophthalmol.

[13] Wiznia RA (1982). Natural history of idiopathic preretinal macular fibrosis. Ann Ophthalmol.

[14] Tan HS, Mura M, de Smet MD (2009). Iatrogenic retinal breaks in 25-gauge macular surgery. Am J Ophthalmol.

[15] Kamizuru H, Ogura Y (1995). Incidence of peripheral retinal degeneration in eyes with idiopathic macular hole. Jpn J Clin Ophthalmol.

[16] Zhang J, Li Y, Zhao X, Cai Y, Yu X, Lu L (2015). Relationship between full-thickness macular hole and retinal break/lattice degeneration. Eye Sci.

[17] Essex RW, Kingston ZS, Moreno-Betancur M, Shadbolt B, Hunyor AP, Campbell WG, et al (2016). The Effect of Postoperative Face-Down Positioning and of Long- versus Short-Acting Gas in Macular Hole Surgery. Results of a Registry-Based Study. Ophthalmology.

[18] Iezzi R, Kapoor KG (2013). No face-down positioning and broad internal limiting membrane peeling in the surgical repair of idiopathic macular holes. Ophthalmology.

[19] Elborgy ES, Starr MR, Kotowski JG, Chehade JEA, Iezzi R (2018.). No face down positioning surgery for the repair of chronic idiopathic macular holes. Retina.

[20] Hu Z, Xie P, Ding Y, Zheng X, Yuan D, Liu Q (2016). Face-down or no face-down posturing following macular hole surgery: a meta-analysis. Acta Ophthalmol.

